# Supplementary material for: Eukaryotic association module in phage WO genomes from Wolbachia
Source: Nat Commun. 2016 Oct 11;7:13155. doi: 10.1038/ncomms13155 (PMC5062602; doi:10.1038/ncomms13155)
Supplement: Supplementary Information — Supplementary Figures 1-4, Supplementary Tables 1-4 [file ncomms13155-s1.pdf]

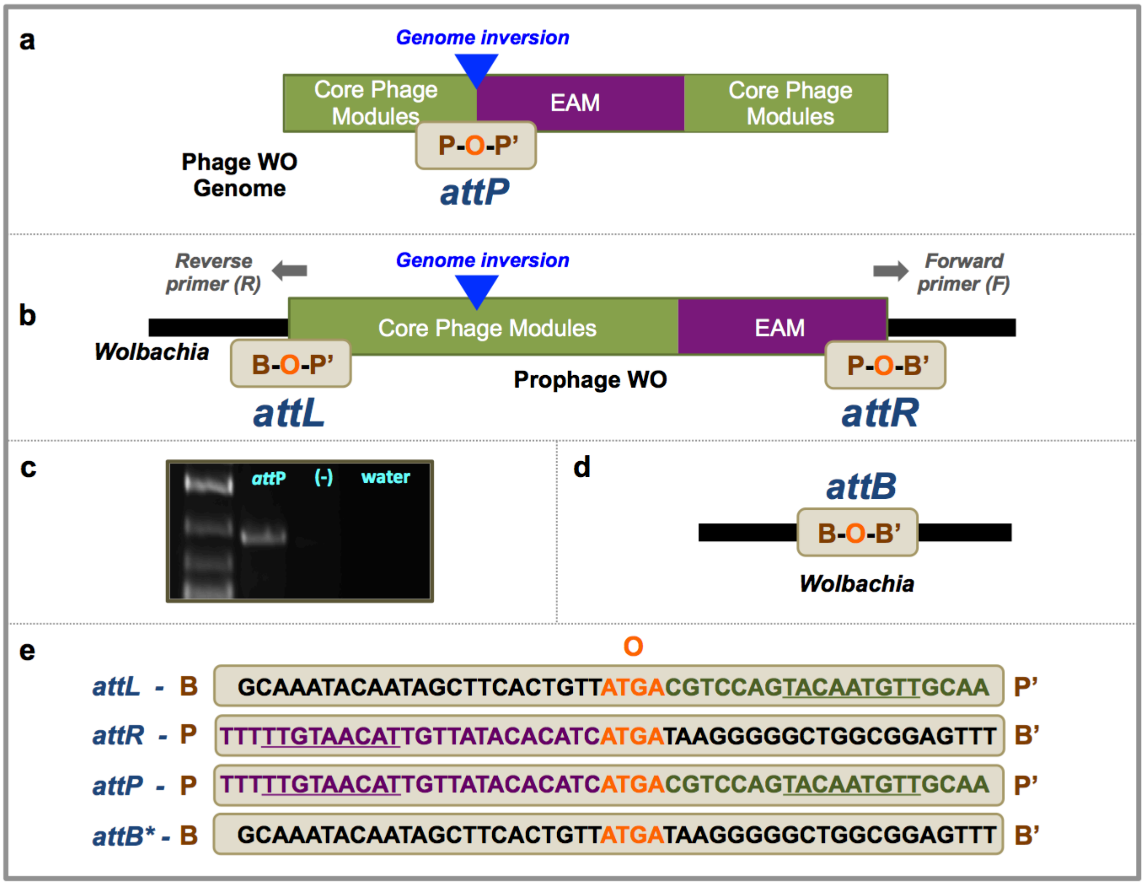

Supplementary Figure 1 | Sequencing reveals the phage, prophage and bacterial *att* sites for WOVitA1. (**a**) The complete phage WOVitA1 genome encodes a eukaryotic association module (EAM, purple) and phage attachment site (*attP*) embedded within core phage structural modules (green). (**b**) The entire phage WO genome, including all core phage modules and the EAM, is inverted (blue triangles) and integrated into the *Wolbachia* chromosome as prophage WO. Attachment sites are designated as BOP' (*attL*) and POB' (*attR*) with B representing 'bacterial' and P representing 'phage'-associated nucleotides. Gray arrows indicate the direction of PCR primers used to amplify the (**c**) phage attachment (*attP*) site, designated as POP' in the linear WOVitA1 genome. (**d**) The bacterial attachment site (*attB*) is designated as BOB' on the *Wolbachia* chromosome. (**e**) All four *att* sites share a common region, O. Underlined nucleotides represent an inverted repeat region. *The *attB* site was predicted based on the *attL* and *attR* sequences. A BLASTN search of this sequence identified the putative *attB* site as a non-coding, repetitive sequence in closely related *Wolbachia* taxa lacking the WOVitA1 infection (e.g., *w*Au, *w*Mel, and *w*Ri).

a

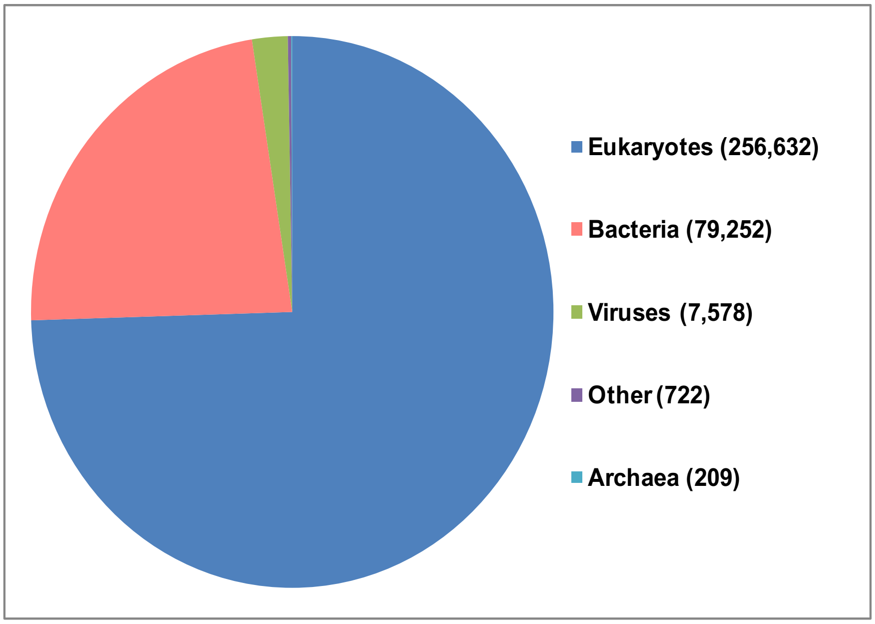

b

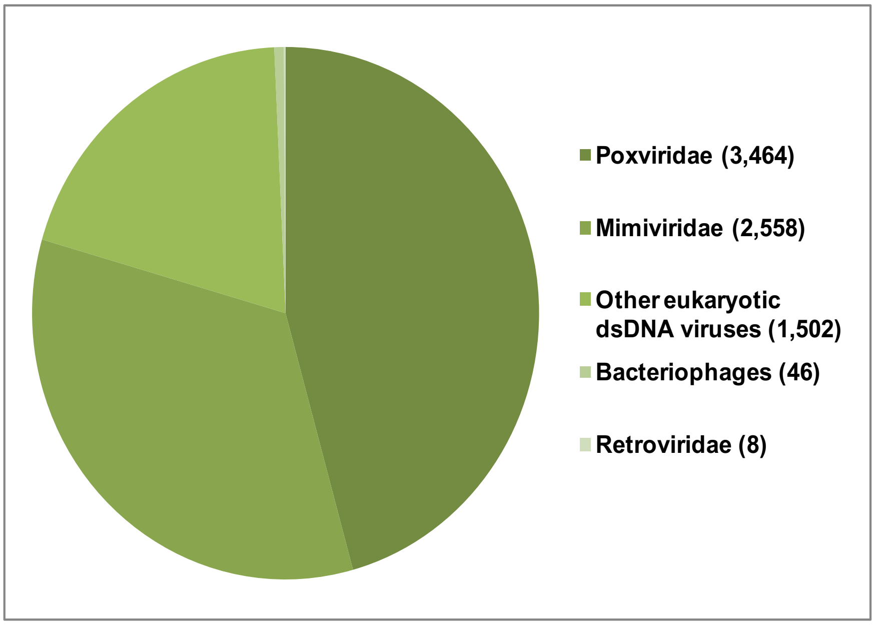

Supplementary Figure 2 | Ankyrin repeat domain. (**a**) The ankyrin domain is most commonly associated with eukaryotes as determined by a taxonomic search of the domain in NCBI's protein database. "Other" refers to synthetically-engineered constructs and metagenomic sequences. (**b**) Of the viral proteins that contain an ankyrin repeat, the majority are associated with either *Poxviridae* or *Mimiviridae*. This analysis does not include prophage regions within bacterial chromosomes.

a

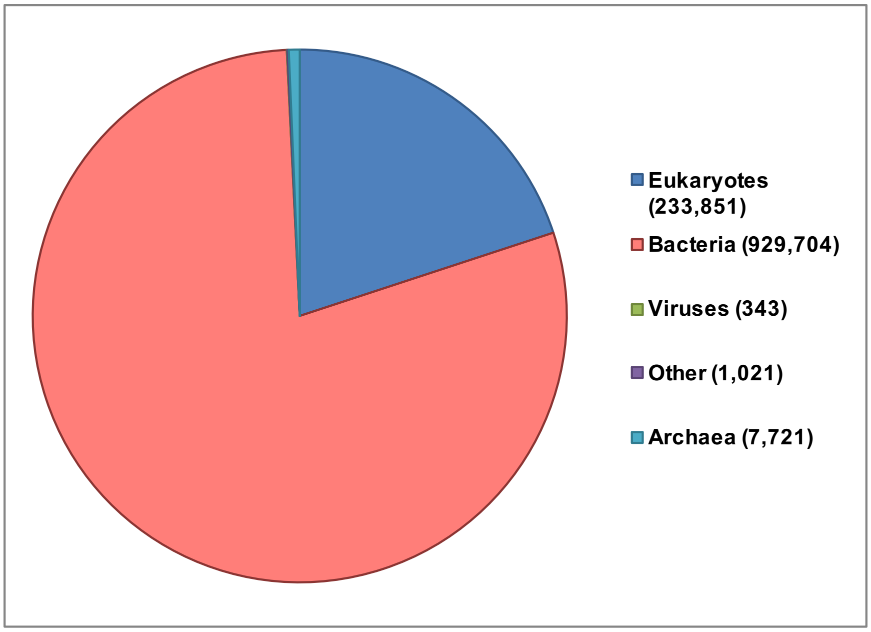

b

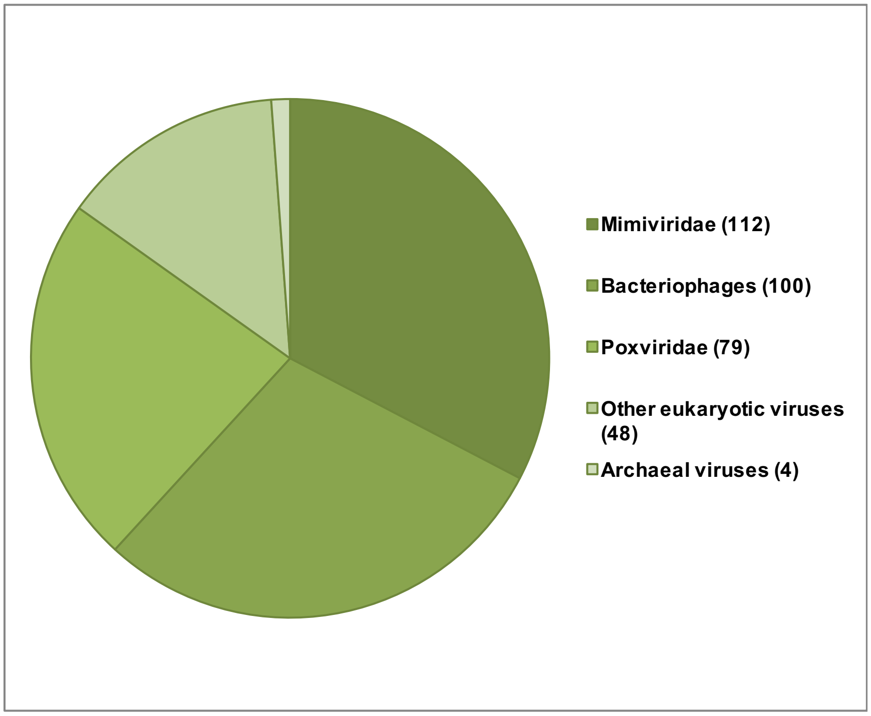

Supplementary Figure 3 | TPR domain. (**a**) The TPR domain is most commonly associated with bacteria as determined by a taxonomic search of the domain in NCBI's protein database. "Other" refers to synthetically-engineered constructs and metagenomic sequences. (**b**) The majority of viral proteins are associated with *Mimiviridae*. This analysis does not include prophage regions within bacterial chromosomes.

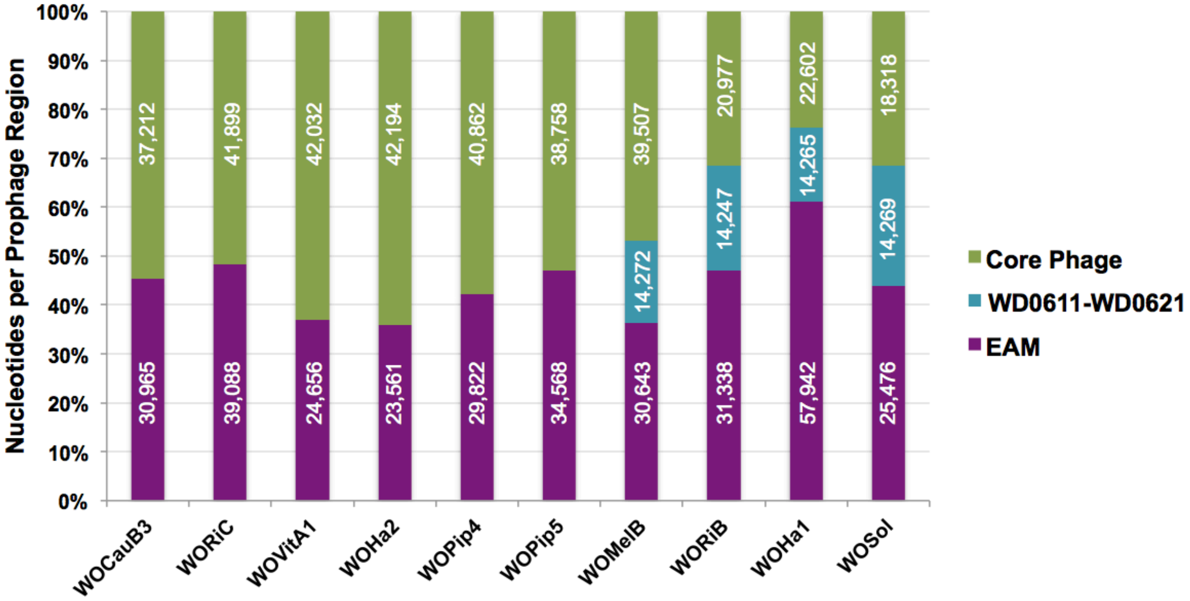

Supplementary Figure 4 | Prophage WO dedicates nearly half of its genome to eukaryotic host association. This chart shows the relative amount of nucleotides comprising the EAM, conserved WD0611-WD0621 cluster, and Core Phage Regions for ten prophage WO haplotypes. The absolute number of nucleotides is labeled in white. The Core Phage Region is defined as the region from recombinase to patatin.

Supplementary Table 1 | *Wolbachia* and phage WO nomenclature. Each *w* refers to a specific *Wolbachia* strain followed by an insect identifier. Each phage haplotype is indicated with (1) a WO- prefix, (2) insect identifier, and (3) specific haplotype identifier (1-5 or A, B, C). For example, WOVitA1 is phage WO haplotype #1 infecting the *Wolbachia* strain, wVitA. (*) *w*CauB is native to *Cadra cautella* and was transferred to *Ephestia kuehniella* prior to sequencing phage particles.

PHAGE WO	*WOLBACHIA*	INSECT
WOAlbB	*w*AlbB	*Aedes albopictus*
WOAnaC	*w*Ana	*Drosophila ananassae*
WOAu	*w*Au	*Drosophila simulans* str. Au
WOBol1-b	*w*Bol1-b	*Hypolimnas bolina*
WOCauB2	*w*CauB*	*Ephestia kuehniella*
WOCauB3	*w*CauB*	*Ephestia kuehniella*
WODi	*w*Di	*Diaphorina citri*
WOOb	*w*Ob	*Operophtera brumata*
WOPip1	*w*Pip	*Culex pipiens quinquefasciatus* str. Pel
WOPip2	*w*Pip	*Culex pipiens quinquefasciatus* str. Pel
WOPip3	*w*Pip	*Culex pipiens quinquefasciatus* str. Pel
WOPip4	*w*Pip	*Culex pipiens quinquefasciatus* str. Pel
WOPip5	*w*Pip	*Culex pipiens quinquefasciatus* str. Pel
WOPipJHB	*w*PipJHB	*Culex pipiens quinquefasciatus* str. JHB
WOPipMol	*w*PipMol	*Culex molestus*
WORiC	*w*Ri	*Drosophila simulans* str. Riverside
WOSuziC	*w*Suzi	*Drosophila suzukii*
WOVitA1	*w*VitA	*Nasonia vitripennis*
WOVitA2	*w*VitA	*Nasonia vitripennis*
WOVitB	*w*VitB	*Nasonia vitripennis*
	*w*Bm	*Brugia malayi*
	*w*Cle	Cimex lectularius
	*w*Oo	*Onchocerca ochengi*

Supplementary Table 2 | Comparative genomics of prophage WO. All intact prophage WO genomes contain a bacteriophage-like region (termed the "Core Phage Region") either adjacent to or flanked by a EAM region, listed with corresponding locus tags. Locus tags followed by (T) indicate that they are flanking transposases and may or may not be part of the prophage region. The (WD0611-WD0621)-like region represents a highly conserved cluster of bacterial genes with a broad range of metabolic function and transport potential. na, not applicable.

PROPHAGE GENOME	CORE PHAGE REGION	EAM REGION	(WD0611-WD0621)-like
WOVitA1	gwv_1104-gwv_1156	gwv_1089-gwv_1103	na
WOVitA2	gwv_426-gwv_459	gwv_458-gwv_472; gwv_484-gwv_496	gwv_473-gwv_483
WOVitA4	gwv_146-gwv_175	gwv_131-gwv_145(T); gwv_176-gwv_178	na
WOCauB2	gp1-gp45	gp46-*(partial sequence)*	na
WOCauB3	gp1-gp44	gp45-GF2gp25	na
WOSol	So0001-So0022	wSo0003(T)-wSo0014; So0023-So0025; So0026-wSo0028(T)	wSo0015-wSo0026
WOMelA	WD0259-WD0288	WD0289-WD0296(T); WD0253(T)-WD0258	na
WOMelB	WD0634-WD0647(T); WD0563(T) - WD0604	WD0605-WD0610; WD0622-WD0633	WD0611-WD0621
*w*Mel WO-Island	na	WD0507-WD0514	na
WOPip1	WP0236(T)-WP0272	WP0273-WP0293	na
WOPip2	WP0297-WP0322	WP0294-WP0296	na
WOPip3	WP0323-WP0342	WP0343(T)-WP0354	na
WOPip4	WP0411-WP0455	WP0405(T)-WP0410; WP0456-WP0465	na
WOPip5	WP1294(T)-WP1340	WP1289(T)-WP1293; WP1341-WP1352(T)	na
WORiA	wRi_012450-wRi_012680(T)	na	na
WORiB	wRi_005400-wRi_005660	wRi_005310(T)-wRi_005390; wRi_005670-wRi_005720; wRi_005860-wRi_005930(T)	wRi_005730-wRi_005830
WORiB(2)	wRi_010060-wRi_010320	wRi_009980(T)-wRi_010050; wRi_010330-wRi_010380; wRi_010500-wRi_010580(T)	wRi_010390-wRi_010490
WORiC	wRi_006880-wRi_007230(T); wRi_007550-wRi_007660(T)	wRi_006620-wRi_006870	na
WOHa1	wHa_02360-wHa_02620	wHa_02010(T)-wHa_2050; wHa_02170-wHa_02350; wHa_02630-wHa_02760(T)	wHa_02060-wHa_02160
WOHa2	wHa_03390-wHa_03840	wHa_03850-wHa_03930	na
WONo1	wNo_01400-wNo01060	wNo_01000(T)-wNo_01050(T)	na
WONo2	wNo_07170(T)-wNo_07380(T)	na	na
WONo3	wNo_09000-wNo_09120	wNo_09130-wNo_09150(T)	na
WONo4	wNo_10070(T)-wNo_10280	wNo_10290-wNo_10350	na
*w*No WO-Islands	na	wNo_01980-wNo_1990; wNo_2110-wNo_02200; wNo_05070-wNo_05150(T)	wNo_2000-wNo_2100

Supplementary Table 3 | Phage WO EAM furin cleavage sites. Genes with predicted furin cleavage sites, indicative of potential host-induced protein modification, were identified within every prophage WO EAM.

GENOME	EAM FURIN CLEAVAGE
WOCauB2	*(partial sequence)*
WOCauB3	gp45, GF2gp18-GF2gp22
WOSol	wSo0011, So0023, So0025
WOMelA	WD0257-WD0258
WOMelB	WD0610, WD0630-WD0632
*w*Mel WO-Island	WD0512-WD0514
WOPip1	WP0280, WP0283, WP0290, WP0292-WP0293
WOPip2	WP0294, WP0319-WP0320
WOPip3	WP0338
WOPip4	WP0407, WP0410, WP0428, WP0433, WP0457, WP0460, WP0462-WP0463
WOPip5	WP1291, WP1341, WP1344, WP1346, WP1349, WP1351
WORiA	(haplotype does not have an EAM)
WORiB	wRi_005330, wRi_005360, wRi_005670, wRi_005720
WORiB(2)	wRi_009990, wRi_010020, wRi_010330, wRi_010380, wRi_010570
WORiC	wRi_006630-wRi_006660, wRi_006740
WOVitA1	gwv_1095
WOVitA2	gwv_464, gwv_484, gwv_489, gwv_491-gwv_495
WOVitA4	gwv_134, gwv_141-gwv_142, gwv_144-gwv_145
WOHa1	wHa_02170, wHa_02200, wHa_02290, wHa_02350, wHa_02690, wHa_02730
WOHa2	wHa_03920
WONo1	wNo_01030, wNo_01060
WONo2	(haplotype does not have an EAM)
WONo3	wNo_09000, wNo_09080, wNo_09140
WONo4	wNo_10290, wNo_10320-wNo_10340
*w*No WO-Islands	wNo_01990, wNo_02030, wNo_02070, wNo_02120, wNo_02130, wNo_05080-wNo_05090, wNo_05110-wNo_05130

Supplementary Table 4 | The phage WO PRANC domain shares amino acid homology with multiple eukaryotic host peptides. The PRANC domain in WOVitA1's gwv_1092 shares homology with multiple insect hosts. The best BLASTP hit for each species is listed above with E-value, query coverage and identity.

EUKARYOTIC HOMOLOG	ACCESSION	E-VALUE	QUERY COVERAGE	IDENTITY
Microplitis demolitor	XP_014298115.1	8.00E-43	75%	49%
Nasonia vitripennis	XP_003426146.1	5.00E-23	76%	37%
Glypta fumiferanae	AKD28025.1	4.00E-21	71%	39%
Trichogramma pretiosum	XP_014232168.1	2.00E-16	73%	33%
Ceratosolen solmsi marchali	XP_011505281.1	8.00E-15	69%	31%
Copidosoma floridanum	XP_014206311.1	3.00E-12	58%	32%
Diaphorina citri	XP_008470724.1	9.00E-10	49%	31%
